# Driving forces of digital transformation in chinese enterprises based on machine learning

**DOI:** 10.1038/s41598-024-56448-w

**Published:** 2024-03-14

**Authors:** Qi-an Chen, Xu Zhao, Xinyi Zhang, Zizhe Jiang, Yuxuan Wang

**Affiliations:** 1 Surrey International Institute, Dongbei University of Finance and Economics, Dalian, 116025 Liaoning People’s Republic of China; 2 School of Economics and Business Administration, Chongqing University, Chongqing, People’s Republic of China

**Keywords:** Digital transformation, Machine learning, Predictive effect, TOE theory, Information technology, Socioeconomic scenarios, Sustainability

## Abstract

With advanced science and digital technology, digital transformation has become an important way to promote the sustainable development of enterprises. However, the existing research only focuses on the linear relationship between a single characteristic and digital transformation. In this study, we select the data of Chinese A-share listed companies from 2010 to 2020, innovatively use the machine learning method and explore the differences in the predictive effects of multi-dimensional features on the digital transformation of enterprises based on the Technology-Organization-Environment (TOE) theory, thus identifying the main drivers affecting digital transformation and the fitting models with stronger predictive effect. The study found that: first, by comparing machine learning and traditional linear regression models, it is found that the prediction ability of ensemble earning method is generally higher than that of tradition measurement method. For the sample data selected in this research, XGBoost and LightGBM have strong explanatory ability and high prediction accuracy. Second, compared with the technical driving force and environmental driving force, the organizational driving force has a greater impact. Third, among these characteristics, equity concentration and executives’ knowledge level in organizational dimension have the greatest impact on digital transformation. Therefore, enterprise managers should always pay attention to the decision-making role of equity concentration and executives’ knowledge level. This study further enriches the literature on digital transformation in enterprises, expands the application of machine learning in economics, and provides a theoretical basis for enterprises to enhance digital transformation.

## Introduction

At present, enterprises worldwide are generally facing the challenges and opportunities of digital transformation. With the rapid development of information technology and the popularization and application of the Internet, digital transformation has become a key path for enterprises to enhance their competitiveness and adapt to market demand, while the digital economy has become more and more prominent in the economic field^1^. In 2022, the Cyberspace Administration of China released the Digital China Development Report^2^, stating that the scale of China’s digital economy reached 50.2 trillion yuan in 2022, the total amount of which ranked second in the world, with a nominal year-on-year growth of 10.3%, and the proportion of GDP increased to 41.5%. A number of core businesses of the digital economy, such as electronic information manufacturing, software business, industrial Internet, and agricultural digitization, have seen rapid year-on-year growth, meanwhile, the White Paper on the Development of China’s Digital Economy issued by the China Academy of Information and Communications Technology in 2022^3^ also shows that the average annual growth rate of China’s digital economy since 2012 has been as high as 15.9%, significantly higher than the average GDP growth rate over the same period. And the Digital Economy Report 2021, published by the UNTCD^4^, makes it clear that the United States and China stand out in terms of their ability to participate in and benefit from a data-driven digital economy. These two countries have the world’s highest 5G penetration rates, are home to half of the world’s hyperscale data centers, and account for 94% of the world’s total AI startup funding over the past 5 years, 70% of the world’s top AI researchers, and nearly 90% of the market capitalization of the world’s largest digital platforms. Given this background, more and more scholars have begun to focus on the research field of enterprise digital transformation, exploring the future direction and prospects of enterprise digital transformation^5,6^.

Many studies have been conducted in the academia to address the influencing factors of digital transformation in enterprises. Some of these studies have focused on the impact of technical innovation on digital transformation, such as the use of web platforms^7^, artificial intelligence^8^, big data analytics^9^, and other emerging technologies in enterprise transformation. Meanwhile, some scholars have also analyzed the importance of factors such as organizational structure^10^, leadership thinking^11^, and employee competence^12^ for the success of digital transformation from an organizational perspective. In addition, environmental factors such as market competition, policies and regulations, and industry characteristics have also been included in the research^13^, furthermore, there are also studies that elaborate on the aspects of corporate digital strategy to explore the impact of different strategies on digital transformation^14,15^. Although the existing literature has empirically demonstrated the effects of variables of different characteristic dimensions on digital transformation, these effects are not single effects, but rather there are relationships such as complementary or substitution between individual characteristics, thus forming a compound effect under the combined effect of multiple factors. At the same time, existing studies use the traditional linear regression model, while in practice, the data related to digital transformation does not meet the linear assumption, that is, the variables may be non-linear relationship. As a result, traditional linear regression models often do not fit the data well, and there are limitations in dealing with nonlinear data.

To solve the problem of multiple factors, this paper will adopt the TOE (Technology-Organization-Environment) theoretical model to assess the degree of enterprise digital transformation. The “TOE” theoretical framework was initially proposed to study and comprehensively analyze the influencing factors that interfere with the adoption of innovative technologies by enterprises, and to classify the factors affecting technical innovation into three levels: technology, organization, and environment^16^. Examining the interactions of the three levels of factors within the same theoretical framework allows for a holistic view of the drivers of digital transformation. The technical level includes the application and innovation of existing digital technologies and the degree of knowledge intensity, the organizational level focuses on the organizational structure and governance structure, including the characteristics of the executive team, corporate competence, and financial status; and the environmental level concentrates on external macro factors such as the construction of digital infrastructure and monetary policy. Previous studies have shown that the TOE framework has broad applicability and explanatory power in the study of technology, organization and environment^17^. At present, scholars continue to expand this framework, for example, according to the nature of different enterprises or the specific situation of the industry, proposed new application methods such as TOE-I model or combination with TAM model, and the analysis of data results from many countries has proved the effectiveness and fundamental significance of TOE framework^18–20^. Meanwhile, this paper uses a machine learning model to process the data, which solves the nonlinear, high-dimensional, and large-scale data challenges that arise in the research process,in addition, the machine learning model has stronger predictive ability and adaptability, and can autonomously adjust and optimize according to the changes in the data, which significantly improves the prediction accuracy, and provides richer and more trustworthy prediction information^21^. In summary, this paper analyzes the role of the above set of factors on enterprise digitization through machine learning approach, quantifies the impact of each factor, and conducts a comparative analysis of different driving forces to provide a more accurate way to comprehensively understand the current situation and development trend of enterprise digital transformation, and to provide theoretical guidance and practical suggestions for the development direction of the implementation of digital transformation in the future enterprises.

Compared to the existing literature, the possible marginal contributions of this paper are as follows: first, at the theoretical level, based on the theoretical perspective of the holistic view, it has found that the multiple drivers affecting the digital transformation of enterprises are not a single effect, which not only evaluates and compares the predictive ability of different dimensions of driver characteristics for the digital transformation of enterprises, but also enriches the idea of the configuration perspective. Second, at the methodological level, most of the existing studies are still dominated by causal inference studies based on multiple linear regression, and only a few studies resort to configuration effects and fuzzy set qualitative comparative analysis (fsQCA). Although some scholars have used this method to focus on the composite effects of multiple factors, it is more suitable for explaining the complex nonlinear causal relationship between conditions and results, which is beneficial for qualitative research and cannot quantitatively predict the driving force of digital transformation in enterprises^22–24^. At the same time, considering that the fsQCA method is more suitable for a few easily classified case studies, in order to conduct a more universal predictive analysis of the driving factors of digital transformation in Chinese enterprises, this article selects A-listed companies in various industries in China from 2010 to 2020 as the initial sample, and for the first time, interdisciplinary machine learning methods are used to analyze the factors affecting enterprise digital transformation, constructing a more accurate prediction model for the intensity of enterprise digital transformation, enriched the application of machine learning methods in the field of economics. Third, at the practical level, this paper adopts the TOE model to take the three factors of technology, organization and environment into comprehensive consideration, and adds the benchmark variable. Meanwhile, the single influence and joint effect of each factor are quantified and compared, so as to predict the driving force of Chinese enterprises' digital transformation, and provide a better reference for the future strategy formulation of enterprises’ digital transformation.

## Literature review

### Application of machine learning in the economic field

The field of economics attaches importance to the study of empirical data, and the analysis of empirical data depends on analytical methods. With the innovative use of machine-learning methods, though it is more applied in natural sciences than in social sciences, the powerful learning ability and self-correcting ability of machine learning are very suitable for the quantitative analysis of the causal relationship among variables in the economic field. With more scholars studying and updating machine learning algorithms themselves, machine learning models have greater advantages in terms of analysis speed, accuracy and comprehensiveness of results^25,26^ and its application to the digital transformation of enterprises has begun to thrive. This study examines the application of machine learning in the field of enterprise digital transformation, summarizing as follows: (1) Akbari et al^27^. used Random Forest Regression to study the driving factors of economic and financial integration, concluding that integration is a gradual process. Meanwhile, the combination of Random Forest Regression and evidence theory can effectively improve the efficiency of enterprise financial risk early warning^28^ (2) Kamalov et al^29^. used Logistic Regression (LR), Random Forest Regression (RFR), Multilayer Perceptron (MLP) and Long and Short-Term Memory (LSTM) to analyze and compare the effectiveness that stock prices and stock returns have in predicting stock movements, discovering that the forecast stock price is more advantageous, (3) Nazareth and Reddy^30^ tested the application performance of machine learning in stock market forecast, investment portfolio management, ideal money, exchange market, financial crisis and bankruptcy and insolvency forecast^31^; also used machine learning model to explore the forecast of financial indicators for the return of Chinese stock market. (4) The study of^32^ confirmed that machine learning has a stronger early warning ability for economic crisis than traditional logic models and integration models. Samitas et al^33^. also uses machine learning as an early warning system for the financial crisis. (5) Achakzai and Peng^34^ developed a new machine learning model: Dynamic Integration Selection (DES) to detect fraud in financial statements. (6) Murugan^35^ used cluster-based XG Boost and cluster-based K-nearest neighbor KNN to analyze financial risk. (7) Mashrur et al^36^. stated that machine learning can predict the possibility of default of individuals or enterprises by identifying loan applicants and enterprises with similar characteristics.

### The motivation for digital transformation

The core of digital transformation is to use digital technology to improve the existing organizational mode of enterprise management, fill the “data gap” between different departments of the enterprise, redesign the production and operation structure and management mode, to improve the efficiency of resource allocation and innovate the management mode^37^. Through the study of the driving factors, enterprises can understand the internal and external environment faced in digital transformation, to better carry out the digital transformation.

In recent years, many domestic and foreign scholars have discussed the preliminary factors of digital transformation of enterprises from the aspects of environment, organization, and management. Existing scholars have multiple dimensions of motivation for digital transformation of enterprises: (1) Technical motivation. Digital skills directly or indirectly affect digital transformation^38^. The individual investment in IT technology cannot produce the expected results. To have a positive impact on digital transformation, it is necessary to combine IT infrastructure with other capabilities of the company to further develop relevant transformation strategies^39^. (2) Organizational motivation. Both digital strategy and organizational ability have positive effects on digital transformation of enterprises^40,41^. (3) Manager motivation. Compared to other factors such as technology, awareness of managers is the biggest obstacle to digital transformation^42,43^. In addition, Hu et al^44^. concluded that the overseas education and work experience of senior executives were positively correlated with the level of digital transformation of enterprises. (4) The motivation of the digital economy. Li et al^45^. believed that digital economy can support enterprises to attain key elements of digital transformation, digital financial inclusion can also significantly improve digital transformation of enterprises^46^. (5) The motivation for intergenerational inheritance. The intergenerational inheritance of family businesses will promote digital transformation to some extent, but its inhibitory effect is greater than the incentive effect^47^. (6) Enterprise internal factors. In addition to enterprise size^48^, enterprise resources, enterprise capabilities and enterprise spirit affect digital transformation as well^49^. (7) Operating environment motivation. Luo et al^50^. found that the business environment can promote digital transformation of enterprises by attracting high-tech talents and increasing technology investment. (8) Policy motivation. Wang et al^51^. discovered that government support, including government subsidies and tax incentives, had a positive influence on digital transformation of enterprises by alleviating financing constraints, increasing R&D investment and improving risk bearing capacity. Moreover, climate policy^52^ and low carbon strategy^53^ are also influencing factors in digital transformation of enterprises. (9) Human capital motivation. Enterprise digitization not only includes the upgrade of digitization-related hardware assets, but also requires the software support of knowledge and skills of staff^54^. (10) Huang et al^55^. considered the changes in consumer behavior and the experience of several industry backbone enterprises realizing their own transformation through the construction of digital platforms constantly enable other enterprises to embark on the road of transformation. The degree of industry competition^56^ and the development level of regional big data^57^ are also key factors that affecting digital transformation of enterprises.

However, the above motivation studies are mainly based on a certain feature of a single dimension, lacking comprehensive consideration and comparative analysis of digital transformation motivation, and it is difficult to be applied to the whole sample. To solve the interaction and configuration effects of various dimensions, the indicators of each dimension can be classified and discussed. After comparing the similarities and differences of the characteristics of different motivation, this study applies TOE theory^16^ which divide the driving factors that affect digital transformation into technical motivation, organization motivation and environmental motivation. Technical motivation serves as an important support of enterprise digital transformation, incorporating enterprise innovation ability and absorption ability,organization motivation focuses on the enterprise internal governance and structure problems; environmental motivation mainly display in government regulation and market environment, which helps to discuss enterprise digital transformation motivation more comprehensively, with the aim of finding out the key drivers of enterprise digital transformation.

## Methods

### Research design

#### Research methods

Machine learning algorithms rely on traditional statistical and mathematical models to identify patterns and regulations in existing data and make predictions or decisions based on these patterns. This study applies the method of ensemble learning and a method of integrating multiple learners to achieve stronger out of sample generalization ability than a single learner. Referring to the existing literature^27,35^, the study chooses the most advanced Gradient Boosting Regression (GBR) and Random Forest Regression (RFR) method, and advanced ensemble learning methods LightGBM and XGBoost, comparing with multiple linear regression and LASSO in the linear research method. The regression mechanisms of the four methods used in this article are as follows:

Firstly, linear regression. Linear regression is a fundamental regression model that assumes a linear relationship between the dependent variable and the independent variable as Formula 1.

1 $$\begin{array}{c}y={\uptheta }_{0}+{\uptheta }_{1}{x}_{1}+{\uptheta }_{2}{x}_{2}+\dots +{\uptheta }_{n}{x}_{n}+\epsilon \end{array}$$

In Formula 1, $y$ is the dependent variable while ${x}_{1},{x}_{2},\dots {x}_{n}$ are independent variables.${\uptheta }_{0},{\uptheta }_{1},\dots {\uptheta }_{n}$ are model parameters and $\upepsilon$ is an error term. The goal of linear regression is to estimate model parameters by minimizing the sum of squared errors (MSE) as shown in Formula 2.

2 $${\mathop {\min }\limits_{\theta } \frac{1}{m}\sum\limits_{{i = 1}}^{m} {\left( {y^{{\left( i \right)}} - \widehat{{y^{{\left( i \right)}} }}} \right)^{2} } }$$

Among them, $m$ is the number of samples, ${y}^{\left(i\right)}$ is the true value of the i-th sample, $\widehat{{y}^{\left(i\right)}}$ It is the predicted value of the i-th sample. By estimating regression coefficients, new independent variable values can be predicted and the relative importance of different independent variables to the dependent variable can be evaluated.

Secondly, LASSO regression. Lasso regression is an improvement on linear regression that adds an L1 regularization term while minimizing the sum of squared errors, as shown in Formula 3.

3 $$\begin{array}{*{20}c} {\mathop {\min }\limits_{\theta } \frac{1}{m}\sum\limits_{{i = 1}}^{m} {\left( {y^{{\left( i \right)}} - \widehat{{y^{{\left( i \right)}} }}} \right)^{2} } + \alpha \sum\limits_{{j = 1}}^{n} {\left| {\theta _{j} } \right|} } \\ \end{array}$$

Among them, $\mathrm{\alpha }$ is a regularization parameter used to control the complexity of the model, ${\uptheta }_{j}$ is a model parameter other than the intercept term. The purpose of LASSO regression is to prevent overfitting of the model and improve its generalization ability by punishing larger parameter values.

Thirdly, Gradual Boosted Regression Trees (GBR). Progressive gradient regression tree is an ensemble learning method based on tree models, which generates multiple trees through multiple iterations, and then weighted and summed the predicted results of these trees to obtain the final predicted value. The objective function of gradient boosting decision tree is Formula 4.

4 $$\begin{array}{*{20}c} {\mathop {{\text{min}}}\limits_{{{\theta }} } \sum\limits _{{i = 1}}^{m} l\left( {y^{{\left( i \right)}} ,\widehat{{y^{{\left( i \right)}} }}} \right) + \sum\limits _{{k = 1}}^{K} \Omega \left( {f_{k} } \right)} \\ \end{array}$$

Among them, $l$ is the loss function used to measure the difference between the true and predicted values, $\Omega$ is the regularization term used to control the complexity of the tree, and ${f}_{k}$ is the function expression for the k-th tree, and $K$ is the number of trees. The advantage of gradient boosting decision trees is that they can optimize the loss function through gradient boosting, and can handle different types of loss functions, such as square loss, absolute loss, logarithmic loss, etc. The parameter estimation of gradient boosting decision trees can be solved through methods such as gradient boosting or Newton boosting.

Fourthly, Random Forest (RFR). Random forest is an ensemble learning method based on tree models, which generates multiple decision trees through multiple random sampling, and then weights or votes the predicted results of these trees to obtain the final predicted value. The objective function of a random forest is Formula 5.

5 $$\begin{array}{*{20}c} {\mathop {{\text{min}}}\limits_{{{\theta }} } \sum\limits _{{i = 1}}^{m} l\left( {y^{{\left( i \right)}} ,\widehat{{y^{{\left( i \right)}} }}} \right) + \sum\limits _{{k = 1}}^{K} \Omega \left( {f_{k} } \right)} \\ \end{array}$$

$l$,$\Omega$,${f}_{k}$,$K$ have same meaning as in GBR. The advantage of random forest is that it can improve the efficiency and effectiveness of the model through techniques such as parallel computing, self-help, and feature random selection. At the same time, it can handle problems such as missing values and category features. The parameter estimation of random forests can be solved through methods such as self-help or extreme random trees.

Fifth, XGBoost. XGboost is an ensemble learning algorithm based on gradient boosting trees, which can be used for both regression and classification problems. Firstly, it uses an optimization strategy called Extreme Gradient Boosting, which can build and train models on multi-core cpUs in parallel, thus greatly improving the computational speed and efficiency. Secondly, it adds a regularization term, which can control the complexity and overfitting risk of the model. The regularization term includes the number of leaf nodes in the tree, the sum of the squares of the weight of each leaf node (the score value of the leafnode), etc. The loss function is

6 $$\begin{array}{c}L\left(\phi \right)=\sum\limits_{i} l\left({\widehat{y}}_{i},{y}_{i}\right)+\sum\limits_{k} \Omega \left({f}_{k}\right)\end{array}$$

where, $L(\phi )$ represents the loss function, ${\widehat{y}}_{i}$​ represents the predicted value of the first sample in the first iteration (the first tree), ${y}_{i}$ represents the true value, and $\Omega ({f}_{k})$ represents the regular term.

Sixth, LightGBM. LightGBM is a machine learning method based on Gradient Boosting Decision Tree (GBDT). It has the following characteristics: it supports categorical features, and can directly process numerical and categorical data without one-hot coding; It supports histogram optimization, which can reduce the number of traversals of the global data set and improve the speed of decision tree construction. Gradient-based One-Side Sampling can reduce the sampling times of large Gradient samples and improve the generalization ability of the model. Exclusive Feature Bundling can combine unrelated or conflicting features into one feature to reduce feature dimension and computation. Leaf-wise with depth limitation is supported to avoid the problems of over-fitting and premature convergence. The corresponding loss function value of each sample at each leaf node is formulated as follows:

7 $$\begin{array}{*{20}c} {L\left( \phi \right) = \frac{1}{2}\sum\limits_{{i = 1}}^{n} {\left[ {{\text{log}}\left( {\frac{{f\left( {x_{i} } \right)}}{{f\left( {x_{{i + 1}} } \right)}}} \right) + \gamma \sum\limits_{{j = 1}}^{m} {y_{i} } \left( {f\left( {x_{i} } \right) - f\left( {x_{i} } \right)} \right)} \right]} } \\ \end{array}$$

where: $n$ is the number of training samples, $m$ is the number of categories, ${x}_{i}$ is the feature vector of the first sample, ${y}_{i}$ is the category label of the first sample, $\gamma$ is the weight coefficient, $f\left(x\right)$ is the predicted value.

In summary, ensemble learning methods effectively compensate for endogeneity and other shortcomings caused by non-linear relationships and interactions between variables in linear relationships, and thus perform well in out of sample prediction tasks^58^. Therefore, the predictive effect of ensemble learning methods on the intensity of enterprise digital transformation should be better than linear research methods such as multiple linear regression.

#### Model setting

To select a more effective prediction model, the model performance is investigated based on model interpretation power and prediction error. In terms of model interpretation ability, refer to the existing literature^29^, this study adopts the following three indicators: (1) In-sample goodness of fit ${\text{R}}_{\text{Is}}^{2}$, the index is used to evaluate the degree of fitting of machine learning model on training data, measure the model prediction effect of the training set, the higher the advantages of fitting in the sample, the higher the explanatory ability of the model. (2) Out-of-sample goodness of fit ${\text{R}}_{\text{oos}}^{2}$. To overcome the defects of the In-sample goodness of fit that it cannot completely reflect the generalization of the model on the new data, this article further selects the Out-of-sample goodness of fit ${\text{R}}_{\text{oos}}^{2}$ to measure the universality of the model.(3) Explanatory variance ${{\text{EVS}}}_{{\text{oos}}}$. It is used to measure the interpretation degree of the variability of the dependent variable, and can explain the variance, that is, to calculate the variance between the predicted value and the observed value, and then measure the generalization ability of the model from the perspective of the variance.

In terms of model prediction error, according to the existing research^59,60^ , out-of-sample mean squared error ${MSE}_{oos}$ is selected to measure the deviation between the predicted value and the actual value. If the model performs well on the training data but has a high mean squared error on the test data, there may be a problem of overfitting, namely that the model does not adapt well to the new data. Therefore, by calculating the out-of-sample mean-square error, the study can evaluate the performance of the model more comprehensively and determine whether it has good generalization ability. Meanwhile, to avoid the influence of extreme values, the average absolute error ${MAE}_{oos}$ and the absolute median difference ${MedAE}_{oos}$ are also used to improve the prediction accuracy of the model. The implications and calculations of the evaluation indicators are shown in Table 1.

**Table 1:** Model evaluation indicators and calculation methods.

Evaluation indicators	Indicator meaning	Computational formula
${\text{R}}_{\text{Is}}^{2}$	In-sample goodness of fit, in the training set, the model predicts values to the actual observed values	$R_{{Is}}^{2} /R_{{oos}}^{2} = 1 - \frac{{\sum _{{i = 1}}^{n} (y_{i} - \widehat{{y_{i} }})^{2} }}{{\sum _{{i = 1}}^{n} \left( {y_{i} - \bar{y}} \right)^{2} }}$
${\text{R}}_{\text{oos}}^{2}$	Out-of-sample goodness of fit, in the training set, the model predicts values to the actual observed values	
${EVS}_{oos}$	Explanatory variance, in the prediction set, the fit of the degree of variation to the actual observed value	$EVS_{{oos}} = 1 - \left( {var(y - \hat{y})} \right)/\left( {var\left( y \right)} \right)$
${MSE}_{oos}$	Mean squared error, the expected value of the square between the out-of-sample predicted value and the actual value	${MSE}_{oos}=1/n{\sum }_{i=1}^{n}{\left({y}_{i}-\widehat{{y}_{i}}\right)}^{2}$
${MAE}_{oos}$	Average absolute error, the expected value of the difference between the out-of-sample predicted and actual value	${MAE}_{oos}=1/n{\sum }_{i=1}^{n}{\left|{y}_{i}-\widehat{{y}_{i}}\right|}^{2}$
${MedAE}_{oos}$	Absolute median difference, median of the difference between out-of-sample predicted and actual values	${MedAE}_{oos}={\text{median of}}\left|{y}_{i}-\widehat{{y}_{i}}\right|$

Moreover, one of the main advantages of ensemble learning is that the disadvantages of a single model can be reduced by combining multiple underlying models, so it is difficult to capture the interpretation results of a single learner. In this regard, this study uses relative importance and partial dependence graph to make up for the above deficiencies and interpret the practical significance of ensemble learning. Initially, relative importance refers to the relative contribution degree or influence of each factor to the outcome during model fitting. According to the practice of Nazareth and Reddy^30^, given that the rest of the model remains constant, the relative importance of the variable can be obtained by measuring the decrease of the loss function caused by adding a variable to the model. The greater the relative importance is, the stronger the ability of this variable to predict the intensity of the digital transformation of enterprises. Secondly, the partial dependency graph refers to the measurement of the influence of a certain variable on digital transformation of an enterprise, if other features remain unchanged, and then displayed in the form of images to attain more visual features. In addition, it makes the single variable more accurate in predicting the degree of enterprise digital transformation^61^.

### Data sources and variable definitions

#### Data source

In this study, the A-share listed companies from 2010 to 2020 are taken as the initial sample, namely listed companies in Shenzhen Stock Exchange and Shanghai Stock Exchange of China. Company data derives from the Wind and CSMAR databases. In order to exclude the interference of some special observation samples to the prediction results, this study handles the data as follows: (1) Excluding enterprises with abnormal ST, PT and other listing status, avoid the interference with the overall prediction effect because of the abnormal operation of the enterprise itself; (2) Eliminate the samples with serious missing data; (3) The continuous variables in the data are winsorized according to 1% and 99% quantiles to avoid the interference of extreme outliers. Finally, 8310 observed values are obtained, and the yearly distribution of observations is shown in the Table 2.

**Table 2:** Yearly distribution of observations.

Year	Freq	Percent	Cum
2011	230	2.77	2.77
2012	1134	13.64	16.41
2013	1351	16.26	32.67
2014	1420	17.09	49.76
2015	1400	16.85	66.61
2016	541	6.51	73.12
2017	487	5.86	78.98
2018	559	6.72	85.70
2019	642	7.73	93.43
2020	546	6.57	100.00
Total	8310	100.00	

#### Variable definition

This study selects the Digital Transformation Index (Digitaltransindex) in the CSMAR database as the response variable. According to the CSMAR variable, the response variable using the annual report of enterprise digital transformation related word frequency statistics, including artificial intelligence (AI), block chain (BD), cloud computing (CC), big data (BD) and the application of digital technology (ADT) five parts, this measure can effectively reflect the enterprise digital transformation and transformation degree, detailed calculation are listed in the variable table.

According to the theoretical framework of TOE and the existing research on the driving force of enterprise digital transformation, this study selects the driving force characteristics of the model from the following three dimensions: Technical dimension, this study uses Tamayo et al^38^. to select the intensity of R&D expenses and the technical size as the measurement index of innovation ability and absorption ability. Organization dimensions, referring to Li et al^57^., Schoar and Zuo^62^, Chen et al^63^. and Bandiera et al^64^., the study selected senior manager team size (Manager Number), senior executives’ knowledge level (Education Level), senior social capital (Social Network), profitability (ROA), growth (Growth), enterprise value (TobinQ), solvency (Lev), equity concentration (Top Ten Holders Rate), duality of chairman and general manager (Duality), and proportion of independent directors (IndDirector Ratio) and other ten variables to Measure characteristics of organizational drive characteristics. Additionally, referring to the research of Li et al^49^., Luo et al^50^., Wu and Wang^65^, financial support (Financial Support), infrastructure index (Infrastructure Score), monetary policy easing (Monetary Policy), intellectual property protection level (IP Protection), and industry competition pressure (HhiD) are taken as variables to measure the environmental characteristics of media companies.

In addition, the benchmark variable group refers to Li et al^57,66^., Zhao et al^67^. and Hanelt et al^68^., we set up past performance (Past Revenue), cash flow ratio (Cash Flow Ratio), enterprise age (Firm Age), enterprise size (Size), ownership (SOE), etc. As shown in also Table 3.

**Table 3:** Variable definition.

Type of variable	Variable name	Definition
Technical	R&D expenses	R&D investment intensity (ratio of R&D investment to operating income)
	Technical size	Proportion of technicians (technical ratio of technical personnel to total employees)
Organizational	Manager number	Natural logarithm of the total number of managers
	Education level	The education level of the senior executive team is measured, that is, the value of other degrees is 1, the college degree is 2, 3, and the graduate degree is 4. The sum of the weight of the senior executive team is divided by the total number of people to obtain the average number to represent the education level of the senior executive team
	Social network	Measure by the total number of senior executives working in other enterprises in the corresponding year
	Top ten holders Rate	Share ratio of the top ten shareholders
	Duality	Duality = 1, non-duality = 0
	IndDirector ratio	The proportion of the number of independent directors to the total number of the board of directors
	ROA	Return on assets (income/total assets)
	Lev	Total liabilities/Total assets
	Growth	(Operating income for this year/Operating income last year)-1
	TobinQ	(Market value of tradable shares + number of non-tradable shares net assets per share + book value of liabilities)/total assets
Environmental	Financial support	The ratio of the local financial expenditure on science and technology to the public budget revenue
	Infrastructure score	The entropy right method is used to construct the infrastructure application and development indicators supporting the development of digital economy into an infrastructure index, with provincial annual data
	Monetary policy	The annual M2 growth rate for that year
	IP protection	The ratio of the contract amount of the technology market of each province to the GDP of each province in the current year is divided into provincial annual data
	HhiD	The Herfindahl–Hirschman Index of the industry in which the enterprise operates
Benchmark	Past revenue	Natural logarithm of company revenue at the end of the year
	Cash flow ratio	Operating net cash flow/total assets
	Firm age	Company listing years
	Size	Log of the total assets
	SOE	Soes = 1, non-soes = 0
Y	Digitaltransindex	Digital transformation index in the CSMAR database

### Empirical results and the analysis

#### Descriptive statistics

According to Table 4, the average value of Digitaltransindex is 37.7564, and the standard deviation is 11.8132, which indicates the degree of digital transformation of different enterprises is significantly different, and the characteristics of other variables have no outliers, which demonstrates the rationality of the prediction.

**Table 4:** Descriptive statistics.

	Count	Mean	Std	Min	25%	50%	75%	Max
R&D expenses	8310	0.0135	0.0454	0	0	0	0.0086	1.1414
Lev	8310	0.4152	0.1994	0.0080	0.2556	0.4092	0.5668	0.9952
Top ten holders Rate	8310	56.8320	14.7931	3.5880	46.2425	57.0800	67.5600	101.1600
Growth	8310	0.5650	10.8048	− 2.7804	− 0.0185	0.1420	0.4336	865.9082
Past revenue	8310	21.5986	1.4400	17.6185	20.6096	21.4243	22.4161	28.1765
Cash flow ratio	8310	0.05189	0.0790	− 1.4811	0.0098	0.0490	0.0927	0.7060
Size	8310	22.2317	1.2790	19.0811	21.3334	22.0253	22.9195	28.5040
Manager number	8310	1.8457	0.3625	0	1.6094	1.7918	2.0794	3.1781
SOE	8310	0.3715	0.4832	0	0	0	1	1
Technical size	8310	5.0817	5.8048	0	1.9800	3.700	5.9500	137.4500
Financial support	8310	4.2602	0.1735	3.9800	4.1300	4.2500	4.4100	4.4900
Monetary policy	8310	12.1790	2.1343	8.2750	10.3267	12.3200	13.5425	14.8467
HhiD	8310	0.0939	0.1082	0.0147	0.0255	0.0625	0.1213	1
TobinQ	8310	2.2075	1.6017	0.6837	1.2997	1.7449	2.5491	31.4002
ROA	8310	0.0355	0.0666	− 1.2401	0.0130	0.0343	0.0638	0.3657
Firm age	8310	16.3154	5.4486	3	13	16	20	48
IndDirector ratio	8310	37.3790	5.4358	18.1800	33.3300	33.3300	42.8600	80
Education level	8310	3.2629	0.4178	0	3	3.3333	3.5556	4
Social network	8310	18.9966	4.9484	8	15	18	22	48
Duality	8310	0.7344	0.4417	0	0	1	1	1
IP protection	8310	0.0264	0.0463	0.0002	3	0.0083	0.0225	0.1750
Infrastructure Score	8310	0.2035	0.0562	0.1019	0.0032	0.1972	0.2412	0.4794
Digitaltransindex	8310	37.7564	11.8132	23.0205	0.1598	34.0385	46.5364	80.0403

#### The fitting results of the model based on the enterprise digital transformation index prediction

Table 5 lists the prediction results of the models constructed by different ensemble learning methods for the degree of enterprise digital transformation. The results in Column (1) show that the in-sample goodness of fit ${\text{R}}_{\text{Is}}^{2}$ of multiple linear regression, LASSO model and GBR, which are all lower than 0.54. While the results of RFR, XGBoost and LightGBM are high, all higher than 0.9, among which XGBoost has reached 0.9867 and shown that the ensemble learning method has better in-sample fitting effect. In addition, the results of columns (2) and (3) of Table 5 show that the out-of-sample goodness of fit ${\text{R}}_{\text{oos}}^{2}$ and explanatory variance ${\text{EVS}}_{\text{oos}}$ of LightGBM have the highest values, which are 0.7350 and 0.7353 respectively, followed by XGBoost, and the four indexes of the two methods are all higher than 0.72. It illustrates that ensemble learning method can better predict the degree of digital transformation of enterprises. As can be seen from column (4), the out-of-sample mean square errors ${\text{MSE}}_{\text{oos}}$ of XGBoost and LightGBM are smaller than those of the other four methods. Finally, columns (5) and (6) show that XGBoost and LightGBM have lower mean absolute errors ${\text{MAE}}_{\text{oos}}$ (5.3023 and 5.2542) and lower median differences ${\text{MedAE}}_{\text{oos}}$﻿ than the linear regression method. This indicates that the model improvement effect is not obvious after removing the off-bias values.

**Table 5:** Results of model fitting.

	${\text{R}}_{\text{Is}}^{2}$(1)	${\text{R}}_{\text{oos}}^{2}$(2)	${\text{EVS}}_{\text{oos}}$(3)	${\text{MSE}}_{\text{oos}}$(4)	${\text{MAE}}_{\text{oos}}$(5)	${\text{MedAE}}_{\text{oos}}$(6)
Multiple Linear Regression	0.2867	0.2718	0.2718	101.0353	8.1011	6.8265
LASSO	0.2105	0.2264	0.2264	101.3372	8.5779	7.5468
GBR	0.5375	0.4292	0.4292	79.2012	7.0630	5.7444
RFR	0.9335	0.4890	0.4896	70.8983	6.6131	5.3068
XGBoost	0.9867	0.7246	0.7247	46.0911	5.3023	4.2538
LightGBM	0.9169	0.7350	0.7353	44.3444	5.2542	4.3761

In summary, XGBoost and LightGBM in the ensemble learning method have better data fitting effect, so that a research model with more accurate prediction effect can be constructed. This paper will further discuss the driving force and key factors of enterprise digital transformation.

#### Differences in the driving force dimensions of enterprises’ digital transformation prediction ability

To explore the differences in the prediction ability of different driving forces on the strength of enterprise digital transformation, this study refers to Chen^63^, and selects the benchmark models of past performance (Past Revenue), cash flow ratio (Cash Flow Ratio), enterprise age (Firm Age), enterprise size (Firm Size) and ownership (SOE). Then, referring to Bertomeu et al^69^., calculate and compare the predictive performance of different combinations of TOE theoretical models added to the benchmark model. Considering that the research conclusions obtained based on different evaluation indicators are basically the same, this study analyzes the out-of-sample goodness of fit ${\text{R}}_{\text{oos}}^{2}$, and the research results are as shown in Table 6.

**Table 6:** Prediction performance under different combinations of driving forces.

${\text{R}}_{\text{oos}}^{2}$	Multiple Linear Regression (1)	LASSO (2)	GBR (3)	RFR (4)	LightgGBM (5)	XGBoost (6)
Benchmark	0.0095	0.0008	0.0583	0.0578	0.6457	0.6229
Benchmark + Technical	0.2062	0.1857	0.3339	0.3760	0.7073	0.6883
Benchmark + Organizational	0.1030	0.0479	0.2748	0.3396	0.7111	0.6946
Benchmark + Environmental	0.1160	0.0857	0.1886	0.2009	0.6583	0.6275
Benchmark + Technical + Organizational	0.2363	0.1956	0.4071	0.4677	0.7317	0.7093
Benchmark + Technical + Environmental	0.2532	0.2173	0.3498	0.3994	0.7067	0.6977
Benchmark + Organizational + Environmental	0.1666	0.1079	0.3344	0.4011	0.7192	0.7014
Benchmark + Technical + Organizational + Environmental	0.2719	0.2264	0.4291	0.4871	0.7351	0.7265

Firstly, the difference in the predictive ability of a single dimension driving force for the intensity of enterprise digital transformation is considered separately. As shown in the second row of Table 6, the prediction effect is the best when the technical features are added to the benchmark model. Taking LightGBM as an example, the out-of-sample goodness of fit of the model is improved to 0.7073, 0.7111 and 0.6583 after adding the characteristics of technical driving force, organizational driving force and environmental driving force into the benchmark model respectively. Secondly, considering the combination of two different types of motivations, comparing the out-of-sample goodness of fit among different groups in Table 6. It is found that the model with organizational driving force in the combination has the best fitting effect. Finally, when all three driving forces are integrated, LightGBM has the strongest explanatory power, followed by XGBoost. According to the prediction results, enterprises need to pay attention to the improvement of organizational driving forces, such as the proportion of top ten shareholders and the knowledge level of the top management team. At the same time, enterprises need to pay attention to changes in the external business environment, so as to seize the opportunity of profitable policies and improve the intensity of digital transformation. The following section will make a detailed analysis of the differences of single factors based on LightGBM and XGBoost, and put forward more specific suggestions for enterprises.

#### Differential analysis of the prediction ability of digital transformation by key factors under different driving forces

**Figure 1:**
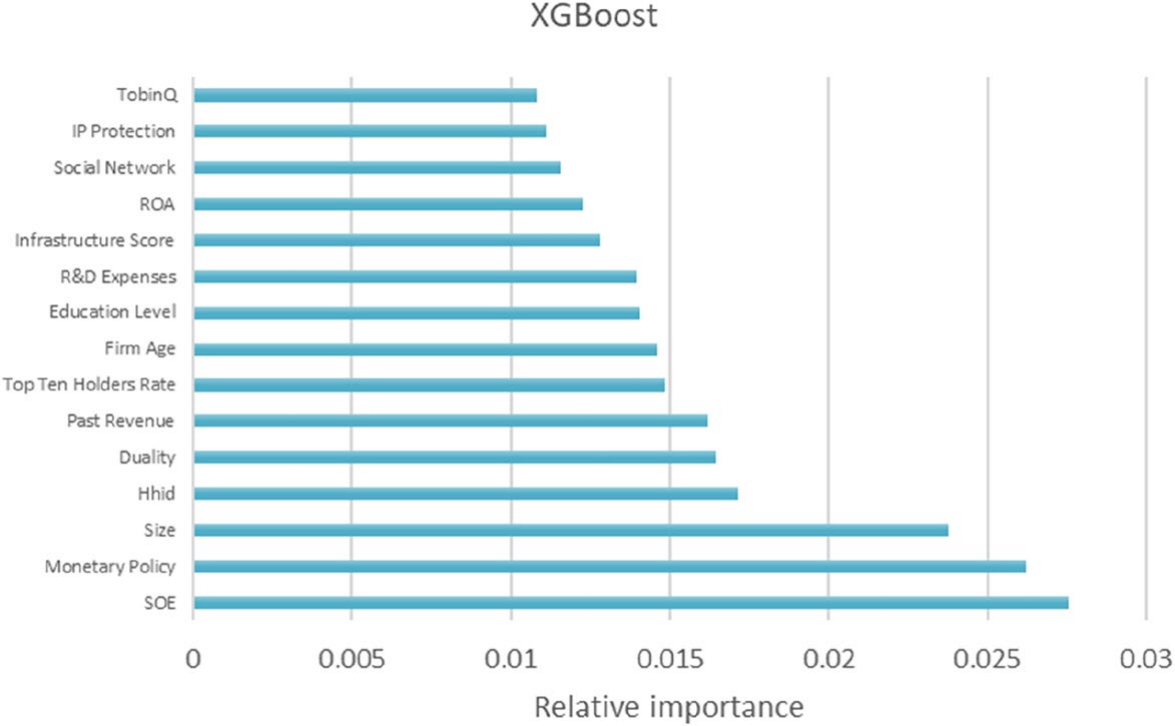
Relative importance ranking based on XGBoost.

**Figure 2:**
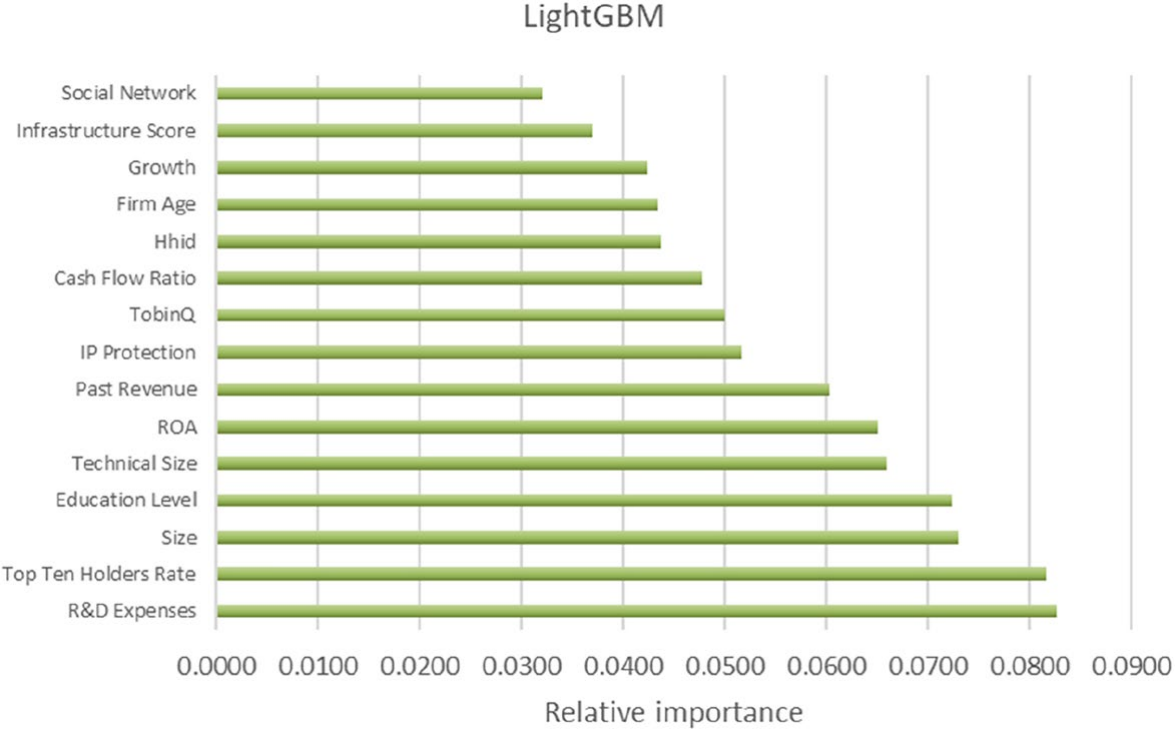
Relative importance ranking based on LightGBM.

Based on the above analysis, the prediction effect of XGBoost and LightGBM is better. Therefore, the two ensemble learning methods of XGBoost and LightGBM are applied to compare the difference in the prediction ability of different variables in the machine learning model for the intensity of enterprise digital transformation by comparing the relative importance. Figures 1 and 2 report the ranking of relative importance of variables, and Table 7 shows the top 15 variables of relative importance in LightGBM and XGBoost prediction methods, which indicates that these characteristics are the key factors affecting the digital transformation of Chinese companies.

**Table 7:** Ranking of relative importance (Top 15).

XGBoost				LightGBM			
Rank	Feature	Dimension	Feature importance	Rank	Feature	Dimension	Feature importance
1	SOE	Benchmark	0.0275	1	R&D expenses	T	0.0827
2	Monetary policy	E	0.0262	2	Top ten holders rate	O	0.0817
3	Size	Benchmark	0.0238	3	Size	Benchmark	0.0730
4	HhiD	E	0.0171	4	Education level	O	0.0723
5	Duality	O	0.0164	5	Technical size	T	0.0660
6	Past revenue	Benchmark	0.0162	6	ROA	O	0.0650
7	Top ten holders rate	O	0.0149	7	Past revenue	Benchmark	0.0603
8	Firm age	Benchmark	0.0146	8	IP protection	E	0.0517
9	Education level	O	0.0141	9	TobinQ	O	0.0500
10	R&D expenses	T	0.0140	10	Cash flow ratio	Benchmark	0.0477
11	Infrastructure score	E	0.0128	11	HhiD	E	0.0437
12	ROA	O	0.0122	12	Firm age	Benchmark	0.0433
13	Social network	O	0.0116	13	Growth	O	0.0423
14	IP protection	E	0.0111	14	Infrastructure score	E	0.0370
15	TobinQ	O	0.0108	15	Social network	O	0.0320

#### Prediction model of the intensity of digital transformation of enterprises by important driving factors

**Figure 3:**
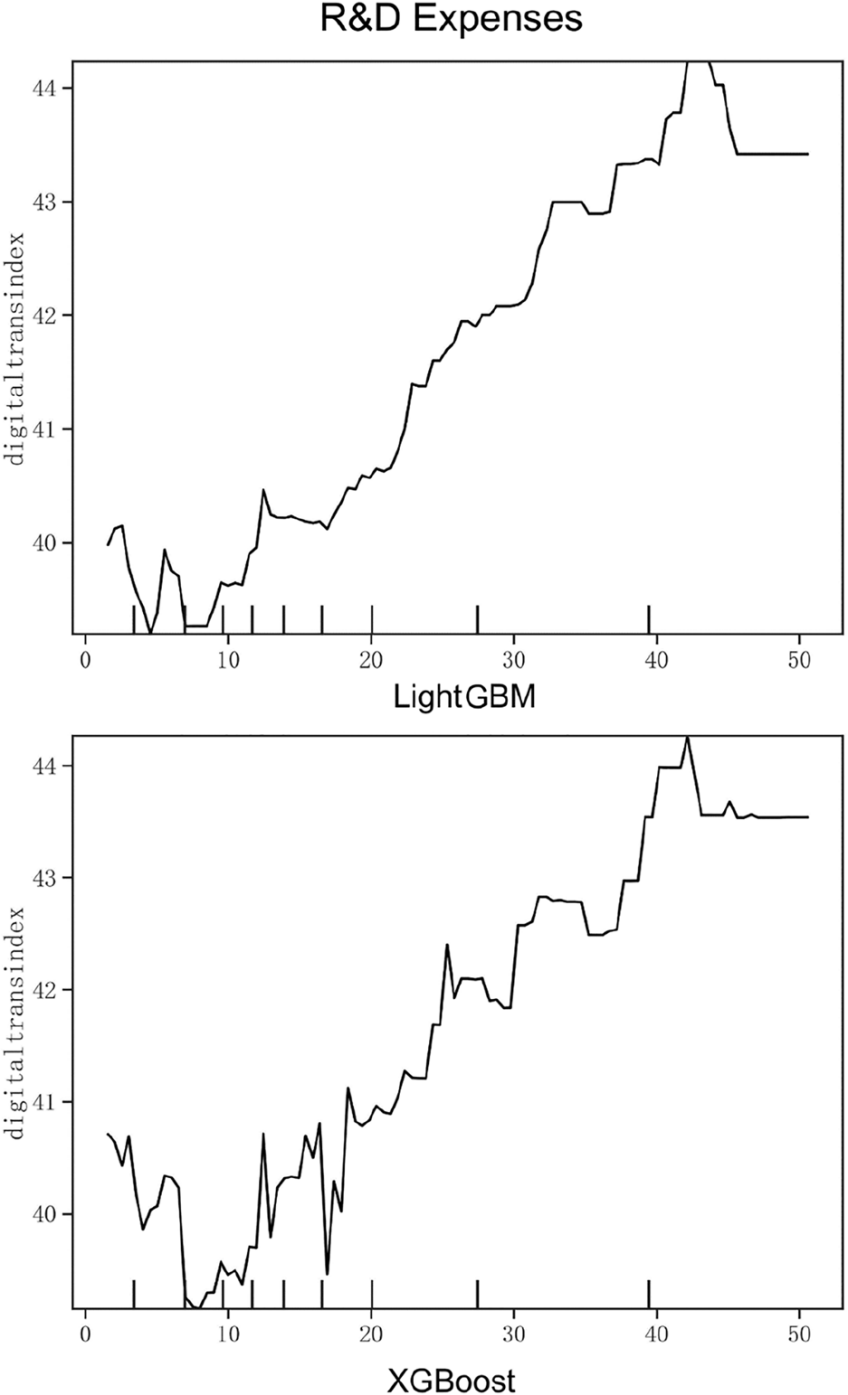
Partial dependence on R&D expenses.

**Figure 4:**
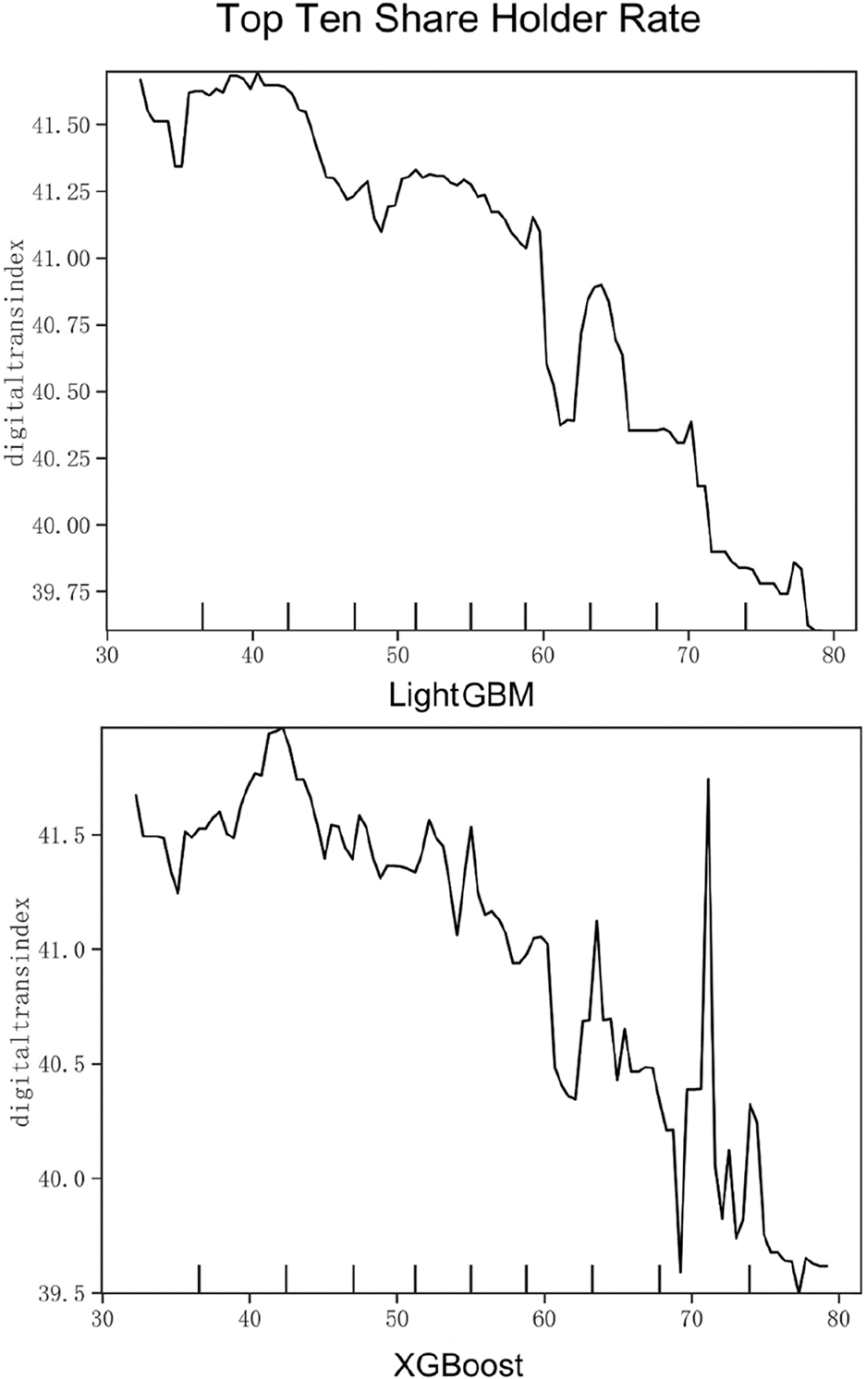
Partial dependence on Top Ten Share Holder Rate.

**Figure 5:**
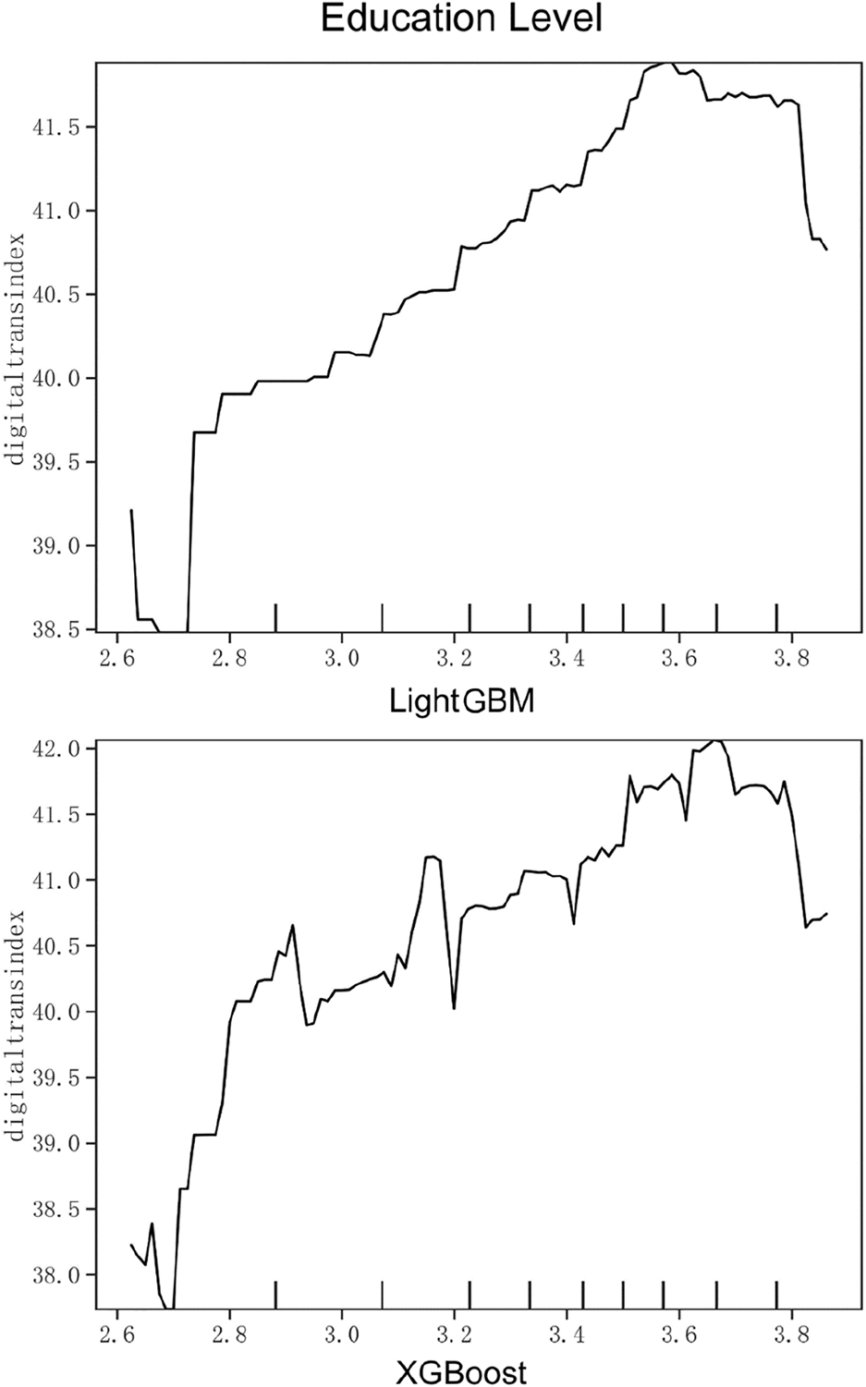
Partial dependence on Education Level.

**Figure 6:**
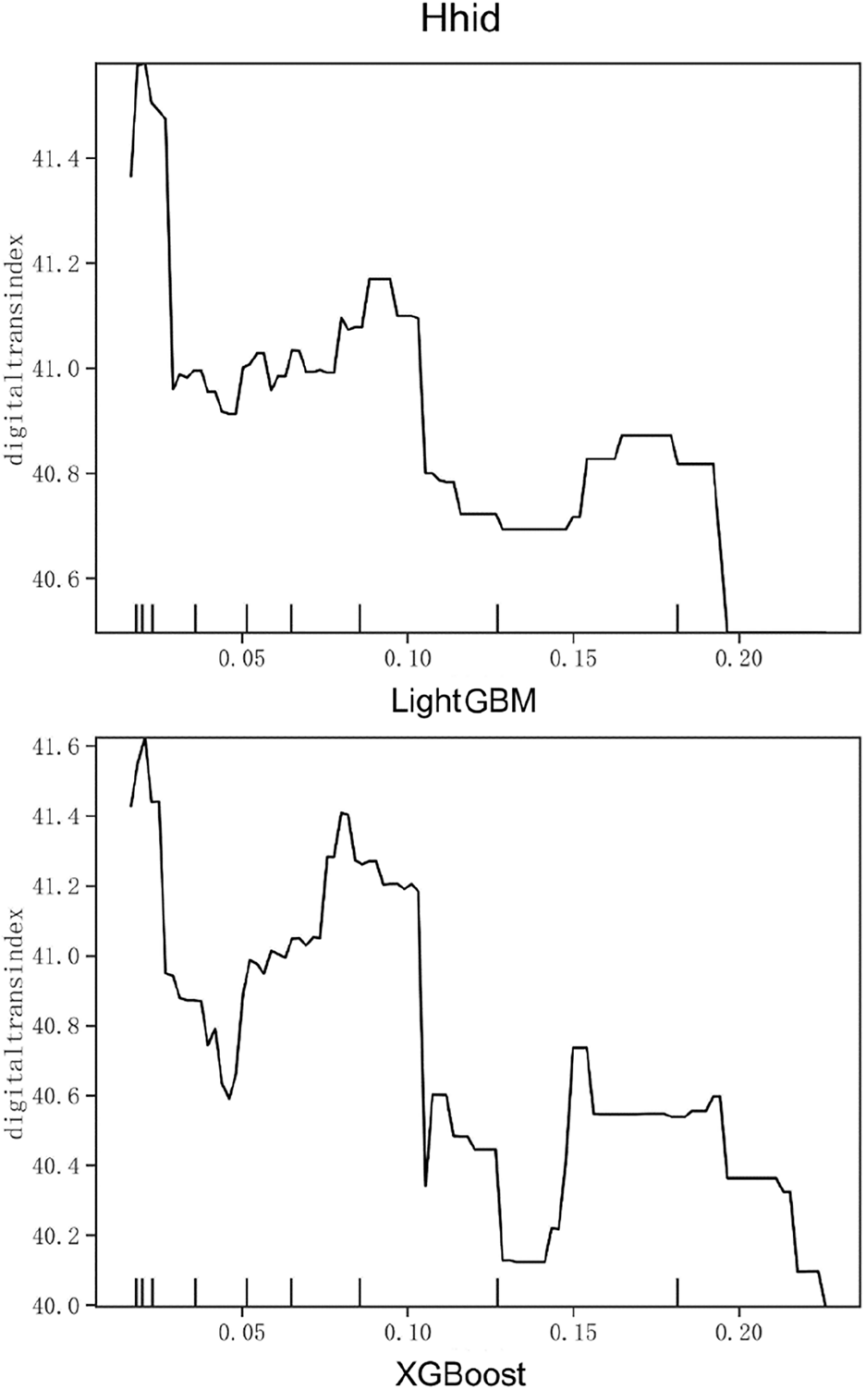
Partial dependence on HhiD.

**Figure 7:**
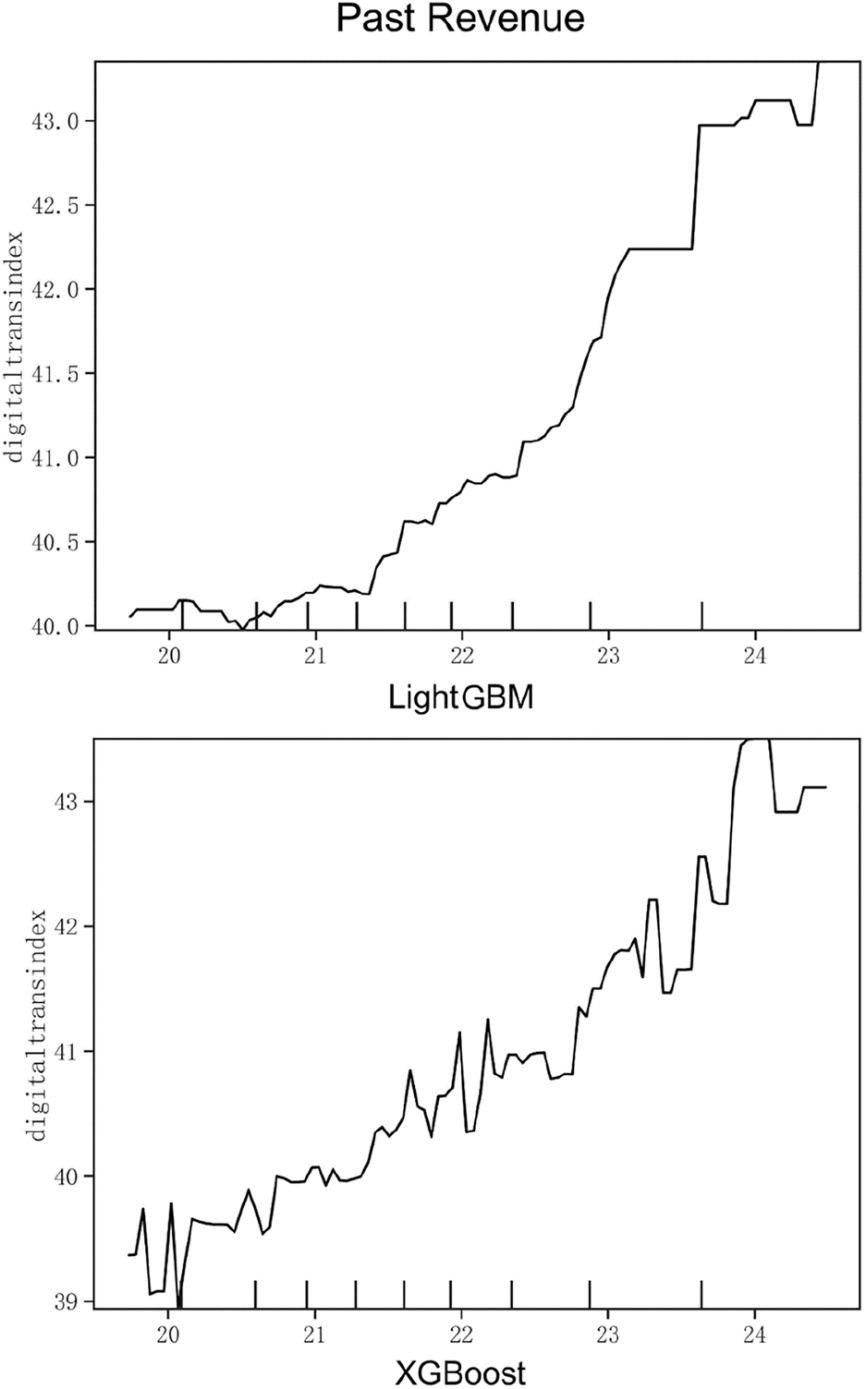
Partial dependence on Past Revenue.

Based on the relative importance and ranking of the variables in Figs. 1 and 2 and Table 7, this study selects innovation ability (R&D Expenses), equity concentration (Top Ten Share Holder Rate), executive knowledge level (Education Level), industry competition degree (HhiD) and past performance (Past Revenue). These variables have higher relative importance in the dimensions of technical, organizational, environmental and benchmark respectively, and have a stronger impact on the digital transformation of enterprises. Meanwhile, they are of universal significance for the digital transformation of companies in different industries. Figures 3, 4, 5, 6 and 7 is partial dependence diagram under LightGBM and XGBoost method.

Figure 3 is partial dependence on R&D expenses. This research selects the R&D investment ratio of enterprises as the proxy variable of innovation capability. As shown in the figure, when the R&D investment of an enterprise is higher than 10%, with the increase of the proportion of investment, the degree of digital transformation also shows a fluctuating upward trend, and reaches the peak when the R&D investment reaches about 42%. When the R&D investment reaches more than 45%, the transformation degree remains at a high level and tends to be flat. R&D investment has the highest relative importance in the technical dimension, indicating that it plays the strongest driving role in the process of digital transformation. Therefore, managers should attach great importance to innovation, not blindly increase R&D expenses, and timely adjust the process of digital transformation.

Figure 4 shows the partial dependence diagram of equity concentration. This paper selects the shareholding ratio of the top ten shareholders as the proxy variable. In general, the fluctuation degree of the image is high, but it still shows a negative correlation trend. When the ratio is around 40%, the degree of transformation is relatively high, and it has a significant decline after reaching 57%. This shows that high equity concentration is not conducive to digital transformation, which is also related to the principal-agent problem within the enterprise. In order to promote the digital transformation and promote the innovation and sustainable development of enterprises, enterprises can introduce more shareholders and stakeholders to make more reasonable decisions.

Figure 5 shows the partial dependence diagram of executives’ knowledge level, which is calculated by assigning and weighting the senior executives’ education level. As shown in Fig. 5, the general trend is that the higher the level of management knowledge, the higher the degree of digital transformation. In particular, the independent variable rises steeply when it reaches 2.7, and then gradually increases. After peaking around 3.6, it begins to decline rapidly. As decision-makers, senior executives with higher education level are better able to accept and implement innovation strategies. At the same time, they also possess professional knowledge and leadership, and can lead the enterprise team to maintain smooth operation in technology research and development, operation and management. Therefore, enterprises should increase the introduction of highly educated talents, optimize the configuration of the top management team, further improve the overall quality and ability level of the top management team, and lay a solid foundation for digital transformation.

Figure 6 shows the partial dependence diagram of industrial competitive pressure, and the proxy variable is the Herfindahl index of the industry in which the enterprise is located. The higher the Herfindahl index, the higher the market concentration, the lower level of the competition. As shown in the figure, it is difficult to describe the relationship between the digital transformation of enterprises and the competitive pressure of the industry with a simple linear relationship. When the Herfindahl index is around 0.02, the degree of digital transformation is the highest. Then it drops sharply, and maintains a relatively stable trend in the range of 0.05–0.10 with a small peak. After reaching 0.18, the digital transformation intensity continues to decline. In general, the greater the competitive pressure in the industry, the higher the degree of digital transformation. Therefore, enterprises in highly competitive industries need to pay attention to the market environment in a timely manner, strengthen the implementation of digital transformation strategy, and establish competitive advantages.

Figure 7 is the partial dependence diagram of the past performance of the enterprise, which natural logarithm of the company’s operating income at the end of the year as the proxy variable. As shown in Fig. 7, the past performance of enterprises shows a positive trend. When it reaches 21.5, the magnitude of the positive impact of past performance on digital transformation gradually becomes larger, accompanied by the appearance of small peaks. Therefore, the annual operating income of the enterprise positively promotes the digital transformation of the enterprise, and the gradient of the influence increases when it reaches a certain value. As a benchmark variable, past performance also ranks high in relative importance among all variables, which proves its universality. Enterprises should first pay attention to the main business, provide funds and operational capacity guarantee for digital transformation, so as to carry out digital reform according to the business situation, and realize the mutual promotion.

#### Robustness test

First, change the training set division method. In the main test of this study, we use 8:2 proportion in random classification to determine the training set and test set, which weakens the randomness to some extent. To evaluate the performance and generalization ability of the model more accurately, K-fold cross-validation is used to replace the training set. The basic principle of K-fold cross-validation is to divide the original data set into K subsets of similar size, where K-1 subsets are used as the training data while the remaining 1 subset is as the validation data. Then, it was repeated K times and a different subset was selected as validation data each time, resulting in the performance evaluation of K models. Usually, we use the average of the results as the final performance evaluation index of the model. The advantage is that it can fully utilize a limited dataset and reduce the variance of model evaluation results. By multiple verifications and averaging, we can more accurately evaluate the performance of the model on different subsets of data, reduce evaluation bias caused by a specific dataset, and provide more reliable evaluation results. The steps of K-fold cross-validation in machine learning are as follows:

1. Divide the original dataset into K subsets of similar size, taking K values of 10.
2. For each subset i (i from 1 to K), take it as the validation set and combine the other K − 1 subset as the training set.
3. In each training session, the model was trained using the training set and evaluated on the validation set.
4. Calculate the evaluation indicators of the model on the validation set, such as accuracy, recall rate, etc.
5. Repeat steps 2 to step 4 to treat the different subsets as validation sets until each subset is used as a past validation set.
6. Average the validation results of K times to obtain the final performance evaluation index of the model.

Based on the process, K-fold cross-validation can obtain more stable evaluation results from repetition of the process to reduce the contingency caused by different data division. Meanwhile, for small data set, K-fold cross-validation can better evaluate the performance of the model, reducing overfitting or underfitting issues caused by a lack of data. As shown in Table 8, after replacing the training and test sets using the K-fold test, the correlation findings compare Table 5 with no change.

**Table 8:** Test of robustness -Panel A.

	${\text{R}}_{\text{Is}}^{2}$(1)	${\text{MAE}}_{\text{oos}}$ (2)	${\text{MSE}}_{\text{oos}}$ (3)	${\text{MedAE}}_{\text{oos}}$(4)	${\text{EVS}}_{\text{oos}}$(5)
Multiple linear regression	0.2752	8.0982	100.9673	7.0795	0.2755
LASSO	0.2114	8.6559	109.9853	7.6287	0.2122
GBR	0.4493	7.0167	76.7933	5.8422	0.4496
RFR	0.5140	6.5405	67.7841	5.4184	0.5150
LightGBM	0.9092	4.968	41.059	3.999	0.739
XGBoost	0.9817	5.156	44.842	4.168	0.716

Second, change the measurement indicators of the intensity of digital transformation. To eliminate outlier or other factors that may affect the uncertainty, this study replaces the measurement indicators of the intensity of digital transformation in enterprises. According to Xiao et al^54^., we use different entry to measure the intensity of digital transformation, eliminating the entry of “digital technology application” from the application level and keeping only basic digital technology level entries “artificial intelligence”, “chain of block technology”, “cloud computing” and “big data technology” . After the total frequency plus 1, we take natural logarithm as the new response variable. The model was re-trained and evaluated using the new response variable. The specific test results are shown in Table 9, the results after the change are consistent with the main test, indicating that the model in this study is robust.

**Table 9:** Test of robustness-Panel B.

	${\text{R}}_{\text{Is}}^{2}$(1)	${\text{R}}_{\text{oos}}^{2}$(2)	${\text{EVS}}_{\text{oos}}$(3)	${\text{MAE}}_{\text{oos}}$ (4)	${\text{MSE}}_{\text{oos}}$ (5)	${\text{MedAE}}_{\text{oos}}$(6)
Multiple linear regression	0.2937	0.2501	0.2501	1.0166	1.6040	0.8389
LASSO	0.1098	0.3851	0.1177	1.1512	1.8871	1.1576
GBR	0.5063	0.1177	0.3852	0.9106	1.3151	0.7561
RFR	0.9266	0.4512	0.4514	0.8546	1.1738	0.6861
LightGBM	0.8870	0.6480	0.6482	0.9486	0.7689	0.6565
XGBoost	0.9797	0.6237	0.6238	1.0142	0.7912	0.6673

## Discussion

Through reviewing the existing literature, it is found that scholars mainly focus on the correlation between a factor of a single dimension and the intensity of enterprise digital transformation, and only make predictions within the sample, lacking comprehensive consideration of the driving force of enterprise digital transformation. In this study, the driving force of enterprise digital transformation is divided into three dimensions: technical driving force, organizational driving force and environmental driving force. By combining and comparing the driving forces of two or three dimensions, the differences in the predictive ability of different dimensions of indicators is listed and the relatively key driving factors are identified. Meanwhile, most existing studies only use traditional econometrics as a tool, which makes it difficult to avoid the interaction between factors and has certain endogeneity issues.

This study takes the relevant data of Chinese A-share listed companies from 2010 to 2020 as samples, discusses the driving force of digital transformation in enterprises, and innovatively uses ensemble learning methods to conduct analysis, which can improve the accuracy of model prediction and enhance its generalization ability. With relative importance ranking and partial dependence graphs, by comparing the fitting effects of adding different dimensional factors to the benchmark model, it is found that technical factors can more effectively and accurately predict the digital transformation behavior of enterprises. This means that in the process of enterprises pursuing digital transformation, technology driving force dominates. Compared with linear methods such as multiple linear regression, the ensemble learning method achieves better performance in high model interpretation ability and less prediction error, among which XGBoost method has the best prediction performance when applied to the samples used in this study. Among many driving force characteristics, equity concentration and knowledge level of executives in the dimension of organizational driving force, and innovation ability in the dimension of technical dimension have the best prediction effect.

Based on the above conclusions, this study proposes the following policy suggestions:

1. For governments, policy support, financial support, technical support, and cooperation opportunities should be provided for enterprises. Financial and tax incentives can be provided to encourage enterprises to invest in the construction of digital technology and information system. Set up special funds to increase the digital infrastructure construction such as network foundation design, cloud computing center and data center, etc. For enhancing the operation performance of enterprises, government can organize professional team and cooperation institutions for technical staff training, encourage higher education institutions, research institutions, and others to participate in the research and innovation work of digital transformation.
2. For the senior management team in enterprises, the strategic goal and path of digital transformation should be clarified. They should strengthen the reserve of high-level talents, and reasonably adjust the proportion of technology research and development. As shown in Fig. 3, when the R&D investment of an enterprise is around 40%, it plays a greater role in promoting the impact of digital transformation. Enterprises should maintain this proportion as much as possible, not blindly invest in R&D, and maximize the transformation. At the same time, enterprises should also assess the risks in the process of digital transformation, take appropriate risk control and response measures, pay attention to the industry policy direction and enterprise value. They can make use of the good economic situation to carry out the layout of transformation. In the process of transformation, performance management is important. Enterprises should actively adjust and innovate their organizational structure, business process and working mode, take the lead in ensuring the stable growth of main business. Then seize the opportunity to carry out digital technology research and development, implement digital transformation strategy, and ensure sufficient funds and organizational stability in the process of transformation.
3. For scholars, continue to focus on the trend of digital transformation. Write professional reports and application cases to provide valuable information and guidance for enterprises and governments, vigorously apply research results to practical scenarios, help enterprises solve practical problems, promote the process of digital transformation, and promote the mutual flow of knowledge and technology.

The limitations of this study are as follows: First, because the data in this study are not randomly sampled, but based on the availability of data, they are not without significant differences from the industry and size distribution of China’s A-share companies, which may lead to the difference in the prediction effect of the potential fitting model. Secondly, the TOE framework cannot cover all the relevant variables and driving factors, for example, the differences in digital transformation modes of different enterprises caused by the characteristics of different industries are not examined. A separate discussion on the degree of digital transformation in each industry will be one of our future research directions. Third, the machine learning methods used in this paper are all black box algorithms. Despite the data robustness test, there is still a risk that the empirical results will be biased due to the errors generated by the algorithm itself. Therefore, it can be considered to combine other analysis methods to make a more comprehensive consideration of enterprise digital transformation.

## Data Availability

The data that support the findings of this study are available from the corresponding author upon reasonable request.

## References

[1] Singhal K, Feng Q, Ganeshan R, Sanders NR, Shanthikumar JG (2018). Introduction to the Special Issue on Perspectives on Big Data. Prod. Oper. Manag..

[2] Cyberspace Administration of China (2022). Digital China Development Report 2022.

[3] China Academy of Information and Communications Technology (2022) White Paper on the Development of China's Digital Economy.

[4] UNTCD (2021). Digital Economy Report 2021. The United Nations Conference on Trade and Development.

[5] Li C, Huo P, Wang Z, Zhang W, Liang F, Mardani A (2023). Digitalization generates equality? Enterprises’ digital transformation, financing constraints, and labor share in China. J. Bus. Res..

[6] Kraus S, Durst S, Ferreira JJ, Veiga P, Kailer N, Weinmann A (2022). Digital transformation in business and management research: An overview of the current status quo. Int. J. Inf. Manag..

[7] Cenamor J, Parida V, Wincent J (2019). How entrepreneurial SMEs compete through digital platforms: The roles of digital platform capability, network capability and ambidexterity. J. Bus. Res..

[8] Huang MH, Rust RT (2018). Artificial intelligence in service. J. Serv. Res..

[9] Manyika J (2011). Big data: The Next Frontier for Innovation, Competition, and Productivity.

[10] Hess T, Matt C, Benlian A, Wiesböck F (2016). Options for formulating a digital transformation strategy. MIS Q. Exec..

[11] Benlian A, Haffke I (2016). Does mutuality matter? Examining the bilateral nature and effects of CEO–CIO mutual understanding. J. Strategic Inf. Syst..

[12] Watson HJ (2017). Preparing for the cognitive generation of decision support. MIS Q. Exec..

[13] Yu F, Du H, Li X, Cao J (2023). Enterprise digitalization, business strategy and subsidy allocation: Evidence of the signaling effect. Technol. Forecast. Soc. Change.

[14] Bharadwaj A, Sawy O, Pavlou P, Venkatraman N (2013). Digital business strategy: Toward a next generation of insights. MIS Q. Manag. Inf. Syst..

[15] Yeow A, Soh C, Hansen R (2018). Aligning with new digital strategy: A dynamic capabilities approach. J. Strategic Inf. Syst..

[16] Tornatzky LG, Fleischer M (1990). The Processes of Technological Innovation.

[17] Hage J (1980). Theories of Organizations: Forms, Process and Transformation.

[18] Zhu K, Kraemer KK, Xu S (2003). Electronic business adoption by European firms: A cross country assessment of the facilitators and inhibitors. Eur. J. Inf. Syst..

[19] Zhu K, Kraemer KK (2005). Post-adoption variations in usage and value of e-business by organizations: Cross-country evidence from the retail industry. Inf. Syst. Res..

[20] Cho J, Cheon Y, Jun JW, Lee S (2022). Digital advertising policy acceptance by out-of-home advertising firms: a combination of TAM and TOE framework. Int. J. Advert..

[21] Ahmed SF (2023). Deep learning modelling techniques: Current progress, applications, advantages, and challenges. Artif. Intell. Rev..

[22] Galeazzo A, Furlan A (2018). Lean bundles and configurations: A fsQCA approach. Int. J. Oper. Prod. Manag..

[23] Miao Z, Zhao G (2023). Configurational paths to the green transformation of Chinese manufacturing enterprises: A TOE framework based on the fsQCA and NCA approaches. Sci. Rep..

[24] Guo J, Fu Y, Sun X (2023). Green innovation efficiency and multiple paths of urban sustainable development in China: Multi-configuration analysis based on urban innovation ecosystem. Sci. Rep..

[25] Pei J, Zhong K, Yu Z, Wang L, Lakshmanna K (2023). Scene graph semantic inference for image and text matching. ACM Trans. ACM Trans. Asian Low-Resour. Lang. Inf. Process..

[26] Chen C, Zhang Z, Wu J, Lakshmanna K (2023). High utility periodic frequent pattern mining in multiple sequences. Comput. Model. Eng. Sci..

[27] Akbari A, Ng L, Solnik B (2021). Drivers of economic and financial integration: A machine learning approach. J. Empir. Financ..

[28] Zhu W, Zhang T, Wu Y, Li S, Li Z (2022). Research on optimization of an enterprise financial risk early warning method based on the DS-RF model. Int. Rev. Financ. Anal..

[29] Kamalov, F., Smail, L. & Gurrib, I. (2020). Forecasting with Deep Learning: S & P 500 index. 422–425. 10.1109/ISCID51228.2020.00102.

[30] Nazareth N, Reddy YVR (2023). Financial applications of machine learning: A literature review. Exp. Syst. Appl..

[31] Zhao C, Yuan X, Long J, Jin L, Guan B (2023). Financial indicators analysis by machine learning: Evidence from Chinese stock market. Financ. Res. Lett..

[32] Liu L, Chen C, Wang B (2022). Predicting financial crises with machine learning methods. J. Forecast..

[33] Samitas A, Kampouris E, Kenourgios D (2020). Machine learning as an early warning system to predict financial crisis. Int. Rev. Financ. Anal..

[34] Achakzai MAK, Peng J (2023). Detecting financial statement fraud using dynamic ensemble machine learning. Int. Rev. Financ. Anal..

[35] Murugan MS (2023). Large-scale data-driven financial risk management & analysis using machine learning strategies. Meas. Sens..

[36] Mashrur A, Luo W, Zaidi NA, Kelly RA (2020). Machine learning for financial risk management: A survey. IEEE Access.

[37] Yi L, Wu F, Xu S (2021). (2021) Research on the performance driving effect of enterprise digital transformation. Secur. Mar. Herald.

[38] Tamayo LAG, Maheshwari G, Odizzio AB, Avilés MH, Delorme CK (2023). Factors influencing small and medium size enterprises development and digital maturity in Latin America. J. Open Innov. Technol. Mark. Complex..

[39] IT and enterprise digital transformation: Findings from Chinese SMEs. Strategic Direction, 39(5),18-20 (2023). 10.1108/SD-03-2023-0036

[40] Luo X, Yu SC (2022). Relationship between external environment, internal conditions, and digital transformation from the perspective of synergistics. Discrete Dyn. Nat. Soc..

[41] Liu S, Yan J, Zhang S, Lin H (2021). Can corporate digital transformation promote input-output efficiency?. Manag. World.

[42] Viet HL, Quoc HD (2023). The factors affecting digital transformation in Vietnam logistics enterprises. Electronics.

[43] Yang L, He X, Gu H (2020). Top management team's experiences, dynamic capabilities and firm's strategy mutation: Moderating effect of managerial discretion. Manag. World.

[44] Hu D, Peng Y, Fang T, Chen CW (2022). The effects of executives’ overseas background on enterprise digital transformation: Evidence from China. Chin. Manag. Stud..

[45] Li R, Rao J, Wan L (2022). The digital economy, enterprise digital transformation, and enterprise innovation. Manag. Decis. Econ..

[46] Guo B, Feng Y, Lin J (2023). Digital inclusive finance and digital transformation of enterprises. Financ. Res. Lett..

[47] Li S, Li X, Wang S, Tong Y (2023). Family firm succession and digital transformation: Promotion or inhibition?. Manag. World.

[48] Yu A, Zhang Y, Liu Y (2023). Research on identification of key influencing factors in the digital transformation of "specialized-elaborative-characteristic-innovative" SMEs-based on the survey of 1625 "specialized-elaborative-characteristic-innovative" SMEs. Econ. Rev..

[49] Li H, Han Z, Zhang J, Philbin SP, Liu D, Ke Y (2022). Systematic identification of the influencing factors for the digital transformation of the construction industry based on LDA-DEMATEL-ANP. Buildings.

[50] Luo Y, Cui H, Zhong H, Wei C (2023). Business environment and enterprise digital transformation. Financ. Res. Lett..

[51] Wang S, Li X, Li Z, Ye Y (2023). The effects of government support on enterprises’ digital transformation: Evidence from China. Manag. Decis. Econ..

[52] Mo Y, Liu X (2023). Climate policy uncertainty and digital transformation of enterprise—evidence from China. Econ. Lett..

[53] Zhao S, Zhang L, An H, Peng L, Zhou H, Hu F (2023). Has China’s low-carbon strategy pushed forward the digital transformation of manufacturing enterprises? Evidence from the low-carbon city pilot policy. Environ. Impact Assess. Rev..

[54] Xiao T, Sun R, Yuan C, Sun J (2022). Digital transformation, human capital structure adjustment and labor income share. Manag. World.

[55] Huang L (2021). The firm's digital transformation and management: Toward a research framework and future directions. J. Manag. Sci. China.

[56] Ma L, Hu H, Li Y (2023). Exploration of digital transformation paths for small and medium-sized enterprises—based on NCA and fsQCA methods. Financ. Account. Mon..

[57] Li Z, Yue T, Jia Y (2023). How does the development of regional big data affect the digital transformation of enterprises?. Mod. Financ. Econ. J. Tianjin Univ. Financ. Econ..

[58] Kleinberg J, Ludwig J, Mullainathan S, Obermeyer Z (2015). Prediction policy problems. Am. Econ. Rev..

[59] Yang C, Abedin MZ, Zhang H, Weng F, Hajek F (2023). An interpretable system for predicting the impact of COVID-19 government interventions on stock market sectors. Ann. Oper. Res..

[60] Khalfaoui R, Jabeur SB, Hammoudeh S, Arfi WB (2022). The role of political risk, uncertainty, and crude oil in predicting stock markets: Evidence from the UAE economy. Ann. Oper. Res..

[61] Friedman JH (2001). Greedy function approximation: A gradient boosting machine. Ann. Stat..

[62] Schoar A, Zuo L (2017). Shaped by booms and busts: How the economy impacts CEO careers and management styles. Rev. Financ. Stud..

[63] Chen Y, Zhou J, Huang J (2023). How does the generosity of enterprises come? Evidence from machine learning. J. Financ. Econ..

[64] Bandiera O, Hansen S, Prat A, Sadun R (2020). CEO behavior and firm performance. J. Political Econ..

[65] Wu Q, Wang X (2021). Financial support, digital inclusive finance and multidimensional poverty alleviation. South China Financ..

[66] Li H, Long H, Wu F (2021). Heterogeneous institutional investors and enterprise digital transformation. Financ. Forum.

[67] Zhao X, Chen Q, Zhang H (2023). Firm investment and financial autonomy: A transaction cost economics and firm lifecycle approach. Manag. Decis. Econ..

[68] Hanelt A, Bohnsack R, Marz D, Marante CA (2021). A systematic review of the literature on digital transformation: Insights and implications for strategy and organizational change. J. Manag. Stud..

[69] Bertomeu J, Cheynel E, Cianciaruso D (2021). Strategic withholding and imprecision in asset measurement. J. Account. Res..

